# TRIM47 Facilitates Osteosarcoma Progression via Destabilising FBP1 and Thus Activation of Wnt/β‐Catenin Pathway

**DOI:** 10.1111/jcmm.70753

**Published:** 2025-08-13

**Authors:** Heng Wang, Xiao Chen, Fengting Nie, Min Zhong, Zhi Fang, Zezhi Qiu, Ling Zhou, Yi Le, Xianpin Wei, Yanyu Liao, Ziling Fang

**Affiliations:** ^1^ Department of Orthopedics the First Affiliated Hospital, Jiangxi Medical College, Nanchang University Nanchang Jiangxi P.R. China; ^2^ Department of Oncology the First Affiliated Hospital, Jiangxi Medical College, Nanchang University Nanchang Jiangxi P.R. China

**Keywords:** fructose 1, 6‐bisphosphatase 1, osteosarcoma, tripartite motif containing 47, tumour progression

## Abstract

Osteosarcoma is a malignant bone tumour with a high rate of disability and mortality in adolescents. Tripartite motif containing 47 (TRIM47) upregulation contributed greatly to carcinogenesis and progression in several tumours, while its role in osteosarcoma (OS) is still unclear and needs further investigation. In this study, we first evidenced that TRIM47 was frequently upregulated in osteosarcoma tissues and cell lines, and the higher TRIM47 expression predicted poor outcomes for osteosarcoma patients. Moreover, TRIM47 depletion impeded cell proliferation, migration, and invasion of osteosarcoma cells, while TRIM47 overexpression elicited opposite effects. Mechanistically, TRIM47 interacted with and accelerated the degradation of fructose 1, 6‐bisphosphatase 1 (FBP1) by inducing its ubiquitination, subsequently activating the Wnt/β‐catenin signalling pathway. Furthermore, knockdown of FBP1 reversed the functions of TRIM47 depletion in OS cells. More notably, our in vivo assays showed that loss of TRIM47 slowed the growth rate of osteosarcoma xenograft tumours. Overall, our data indicated that TRIM47 facilitates OS progression by promoting proteasomal degradation of FBP1, thereby activating the Wnt/β‐catenin pathway, which clarified that targeting the TRIM47‐FBP1‐β‐catenin axis could be a promising approach for treating OS.

**Abbreviations**
CCK‐8 assay: Cell Counting Kit‐8 assay
CHX: cycloheximide
FBP1: fructose 1, 6‐bisphosphatase 1
OS: osteosarcoma
qRT‐PCR: quantitative reverse transcription PCR
TRIM47: tripartite motif 47

## 1 Introduction

Osteosarcoma (OS) is the most common malignant bone tumour in children and adolescents, with a high rate of morbidity and disability [1]. The 5‐year overall survival of osteosarcoma patients without metastasis has improved from 20% to 70% [2], while the prognosis of metastatic OS patients remains low at approximately 20% [3, 4]. Therefore, it is important and urgent to explore the potential mechanisms of OS progression, as well as to identify comprehensive and effective biomarkers for treating OS.

Mounting literature has demonstrated that the tripartite motif‐containing family (TRIMs) proteins belong to the E3 ubiquitin ligase and contribute greatly to a series of pathophysiological processes, including cell differentiation, proliferation, DNA damage repair, and autophagy [5, 6]. Recently, research into the TRIM protein family has focused on its roles in the development and progression of human cancers through the regulation of the ubiquitin‐mediated degradation [7, 8]. Besides, our previous studies have shown that TRIMs elicited a predominant role in tumorigenesis and progression in multiple cancers [9, 10]. Tripartite motif containing 47 (TRIM47), a RING‐type E3 ubiquitin‐protein ligase, was found to be upregulated and correlated with disease progression in several cancers, including gastric cancer, prostate cancer, breast cancer, and glioma [11, 12, 13, 14]. For example, TRIM47 was upregulated in colorectal cancer, and its overexpression facilitated tumorigenesis by inducing the ubiquitination and degradation of SMAD4 [15]. In addition, TRIM47 overexpression conferred endocrine resistance via PKC‐ε/PKD3 stabilisation and thereby activating NF‐κB signalling in breast cancer [16]. However, the detailed functional role of TRIM47 in OS and the underlying mechanism remain unknown.

Here, we firstly reported that TRIM47 was upregulated in OS and positively correlated with malignant behaviours and poor prognosis. Cellular experiments revealed that TRIM47 accelerates the proliferation and invasion of osteosarcoma cells in vitro, and TRIM47 depletion elicited opposite effects. Mechanistically, TRIM47 reduced the protein stability of fructose 1, 6‐bisphosphatase 1 (FBP1), thereby triggering the activation of the Wnt/β‐catenin pathway, ultimately leading to tumorigenesis and progression of osteosarcoma. Therefore, targeting the TRIM47/FBP1 axis may provide new therapeutic targets for treating OS.

## 2 Materials and Methods

### 2.1 Tissue Samples and Ethics Statement

This study had obtained approval from the Ethics Committee of the First Affiliated Hospital of Nanchang University. A total of 60 paired osteosarcoma tissues and corresponding non‐cancerous tissues were collected from the Department of Pathology at our hospital between 2000 and 2023. The eight pairs of fresh osteosarcoma specimens and adjacent tissues were obtained from patients who were diagnosed and treated with surgery alone between 2019 and 2023. The eight fresh specimens were immediately frozen and stored in a liquid nitrogen tank. The related clinical characteristics of the patients were collected and summarised in Table 1 and Table S1. All these patients were required to sign and approve the written informed consent.

**TABLE 1:** Correlation between TRIM47 expression and clinicopathological factors of osteosarcoma patients.

Clinicopathological parameters	*N*	TRIM47 expression (%)		*p*
		Negative	Positive	
	60	23 (38.3)	37 (61.7)	
Age (years)				
< 14	18	7 (38.9)	11 (61.1)	0.956
≥ 14	42	16 (38.1)	26 (61.9)	
Sex				
Male	37	12 (32.4)	25 (67.6)	0.233
Female	23	11 (47.8)	12 (52.2)	
Tumour size (cm)				
< 8	29	15 (51.7)	14 (48.3)	0.039
≥ 8	31	8 (25.8)	23 (74.2)	
Local recurrence				
Yes	12	6 (50.0)	6 (50.0)	0.353
No	48	17 (35.4)	31 (64.6)	
AJCC stage				
IIA	28	16 (57.1)	12 (42.9)	0.005
IIB	32	7 (21.9)	25 (78.1)	
Lung metastasis				
Yes	10	1 (10.0)	9 (90.0)	
No	50	22 (44.0)	28 (56.0)	0.044

### 2.2 Cell Culture and Transfection

The six osteosarcoma cell lines 143B, MG‐63, HOS, U2OS, OS‐732, Saos‐2, and normalised NHOst were purchased from the cell bank of Shanghai Academy of Sciences in China. The cells were cultured with RPMI‐1640 medium (Carlsba Life Technologies, USA) or Dulbecco's Modified Eagle's medium (DMEM, Hyclone, USA) supplemented with 10% fetal bovine serum, penicillin, and streptomycin and incubated in a humidified chamber with 5% CO_2_ at 37°C. Human TRIM47‐targeting siRNAs, TRIM47‐targeting shRNAs, FBP1‐targeting siRNA, and TRIM47 overexpression plasmids were designed and synthesised by Genepharma company (Suzhou, China). The transfection or infection assays were carried out using transfection reagent (Thermo Scientific, R0532, USA) for subsequent experiments as previously described [17].

### 2.3 Immunohistochemistry (IHC) and Western Blotting Analysis

Briefly, formalin‐fixed and paraffin‐embedded osteosarcoma tissue sections were dewaxed with xylene. Upon rehydration and retrieval, all these sections were blocked with 5% BSA for 1 h, then incubated with indicated primary antibodies at 4°C overnight. The next day, sections were stained with diaminobenzidine, followed by counterstaining with haematoxylin and PBS as previously described [18]. The evaluation of staining scores was conducted by two experienced pathologists independently as follows, (0: no staining; 1: weak staining, 2: moderate staining, 3: strong staining) and the percentage of stained cells (negative: 0%; +: 1%–24%; ++: 25%–49%; +++: 50%–74%; ++++: 75%–100%). The final immunohistochemical scores were the products of the positive staining rate and the positive staining area score. We regard 0, 1, 2, 3, and 4 as low TRIM47 expression, and 6, 8, 9, and 12 as high TRIM47 expression. All the osteosarcoma patients signed the informed consent for research purposes.

The western blotting experiments were performed as reported previously [19]. Total proteins collected from the indicated tissues and cells were separated by gradient SDS‐PAGE gel and transferred to PVDF membrane. The cropped membranes were subsequently incubated with the antibodies as follows: TRIM47 (1:300, Proteintech, 26885‐1‐AP); FBP1 (1:5000, Proteintech, 68446‐1‐Ig); β‐catenin (1:5000 Proteintech, 51067‐2‐AP); cyclin D1 (1:1000, Abcam, ab239794); c‐Myc (1:1000, Abcam, ab32072); GSK‐3β (1:5000, Proteintech, 68446‐1‐Ig); FOXM1 (1:5000, Proteintech, Ab207298) and β‐actin (1:3500, Cell Signalling Technology, #4967). β‐actin or GAPDH was utilised as an internal control, and Image J software (https://imagej.net/ImageJ) was used to analyse the protein bands.

### 2.4 Quantitative Real‐Time Polymerase Chain Reaction (qRT‐PCR) Assays

The total RNA extracted from tissues and cells was isolated using Trizol Reagents (Invitrogen, Carlsbad, CA, USA) as reported previously [20]. The cDNA was carried out by a First Strand cDNA Synthesis Kit (Invitrogen, Carlsbad, CA, USA). The relative gene expression was analysed using the SYBR Green kit (Bio‐Rad, Hercules, USA) on the Step‐One plus system. The primers of the genes used in our study were illustrated in Table 2. The formula of 2^−ΔΔCT^ was utilised to determine the relative expression of mRNA, and β‐actin was used for normalisation. All these experiments were performed with more than three replicates.

**TABLE 2:** Sequences of PCR primers for various genes.

Gene	Primer	Sequence (5′→3′)
TRIM47	Forward	CAAGAGTGCAGCCGTAGCAGA
	Reverse	TCAGGGACCTGGCTGAGATTC
FBP1	Forward	TTCGACACGGACGTCAACAC
	Reverse	CAGCAATGCCATAGAGGTGC
β‐catenin	Forward	TAATACGACTCACTATAGGG
	Reverse	TAGAAGGCACAGTCGAGG
c‐Myc	Forward	CCTGGTGCTCCATGAGGAGAC
	Reverse	CAGACTCTGACCTTTTGCCAGG
GSK‐3β	Forward	GCAUUUAUCGUUAACCUAA
	Reverse	UUAGGUUAACGA UAAAUGC
CyclinD1	Forward	CCCTCGGTGTCCTACTTCAA
	Reverse	GGGGATGGTCTCCTTCATCT
β‐actin	Forward	CACCCAGCACAATGAAGATCAAGAT
	Reverse	CCAGTTTTTAAATCCTGAGTCAAGC

### 2.5 Cell Viability and Colony Formation Assays

The ability of cell growth was detected by CCK‐8 and colony formation assays according to the methods reported in our literature previously [10]. Upon transfection for 24 h, 1200 cells per sample were seeded onto 96‐well plates, and the cell viability was evaluated at days 0, 1, 2, 3, and 4. Two hours after the CCK‐8 reagent addition, the supernatant was removed, and the absorbance of each well was evaluated at 450 nm in a microplate reader.

For the colony forming assays, indicated 1000 cells per sample were inoculated into 6 well‐plates for 10–14 days until the colonies were visible to the naked eye. The colonies were stained with 4% paraformaldehyde (Solarbio; Beijing, China) containing 1% crystal violet. Image J software was utilised to count the number of colonies. All these biological experiments were performed in triplicate.

### 2.6 Wound Healing and Transwell Invasion Assays

For the wound scratch assay, osteosarcoma cells were seeded in the 6‐well plates and incubated until 90%–95% confluence. Next, sterile 20 μL pipette tips were used to scrape the bottom surface of the plate to form a vertical wound. The cells were captured at the indicated time points after scratching, and the distances between the two edges of the wound were measured for statistical analysis.

For the transwell invasion assays, the Matrigel‐coated (BD Biosciences, Bedford, MA, USA) chambers (pore size: 8 μm; Corning Costar, NY, USA) were used to evaluate cell invasion ability, as we previously performed [21]. The suspension of osteosarcoma cells (HOS and U2OS) transfected with TRIM47 siRNA, TRIM47 overexpression, FBP1 siRNA, TRIM47 siRNA + FBP1 siRNA, and the corresponding negative controls was seeded into the upper chamber. After 48 h, the cells were fixed and stained with 4% paraformaldehyde supplemented with 1% crystal violet solution. The cells passing through the filter were counted and photographed for statistical analysis. All these experiments were performed in triplicate.

### 2.7 Cycloheximide (CHX) Chase Assay

The protein stability of the FBP1 protein was examined by cycloheximide chasing assays as previously described [22]. Cycloheximide reagent (50 μg/mL) was added to the indicated plates for a specific period, and the cell lysates were subjected to western blotting analysis for further analysis.

### 2.8 Co‐Immunoprecipitation (Co‐IP) Assays

The human Flag‐TRIM47 and HA‐FBP1 plasmids were generated and cloned into the pcDNA3.1 vector with a Flag or HA tag as previously described [20]. OS cells were transfected with Flag‐TRIM47 alone or both HA‐FBP1 and Flag‐TRIM47 plasmids, respectively. Then, cell lysates were used for co‐IP with HA or Flag beads, and protein expressions of TRIM47 and FBP1 were determined. Cell lysates were harvested using lysis buffer for IP with proteinase inhibitors (Beyotime Biotechnology). To reduce non‐specific binding, total protein was primarily incubated with 30 μL of protein A/G immunoprecipitate magnetic beads (Millipore, Massachusetts, USA). The primary HA or Flag antibody was added to the protein lysis at 4°C overnight. Then, protein A/G beads were added to each immunoprecipitation mixture for 4 h. Then the protein‐bead complexes were washed with lysis buffer at least 3 times and denatured for western blotting analysis against specific antibodies.

### 2.9 In Vivo Ubiquitination Assay

HOS cells were seeded into a 10 cm plate and transfected with TRIM47 overexpression plasmids or vector. After 48 h, the cells were treated with MG132 for 6 h before being harvested. Then the indicated cells were lysed by RIPA buffer and immunoprecipitated with Ni‐NTA beads. The ubiquitination levels of FBP1 protein were detected using western blotting assays, as we previously described [23].

### 2.10 Immunofluorescence Staining Assays

After cell attachment, HOS cells were washed twice with phosphate‐buffered saline (PBS). The cells were then fixed with 4% paraformaldehyde (PFA) for 15 min at room temperature, followed by permeabilisation with 0.3% Triton X‐100 for 10 min. Non‐specific binding sites were blocked with 5% bovine serum albumin (BSA) for 1 h at room temperature. Subsequently, the cells were incubated with primary antibodies against TRIM47 and FBP1 overnight at 4°C. The following day, the cells were incubated with fluorophore‐conjugated secondary antibodies for 30 min at room temperature in the dark. Cell nuclei were counterstained with 4′,6‐diamidino‐2‐phenylindole (DAPI). Finally, the subcellular localisation of TRIM47 and FBP1 was examined using a Nikon ECLIPSE Ti2 confocal microscope (Tokyo, Japan).

### 2.11 Animal Xenograft Model

The animal experiments were performed and approved by the Animal Care and Use Committee of Nanchang University (No: CDYFY‐IACUC‐202311QR018). 4–5 weeks old BALB/C‐nude female mice were purchased from Shanghai Laboratory Animal Company and divided into two groups by randomisation. 1 × 10^8^ U2OS cells (scramble group and TRIM47 shRNA group) were injected subcutaneously into the right dorsal flanks to construct a mouse xenograft model. The tumour size of the mice was measured every 3 days following the standard as previously reported [24]. Mice were euthanised at 28 days, with xenografts dissected, weighed, and photographed.

### 2.12 Statistical Analysis

All the data were shown as mean ± SEM of three independent experiments for the cellular experiments and represented as the mean ± SD of 6 mice for the tumour growth study in xenograft models. The mean difference among groups was assessed by Student's two‐tailed *t*‐test, ANOVA, and *χ* ^2^ test, respectively. Statistical analysis was performed using SPSS 26.0 software (IBM, USA). *p* < 0.05 was considered statistically significant (*p < 0.05; **p < 0.01).

## 3 Results

### 3.1 TRIM47 is Frequently Upregulated and Positively Associated With Poor Clinical Outcomes of Osteosarcoma Patients

**FIGURE 1:**
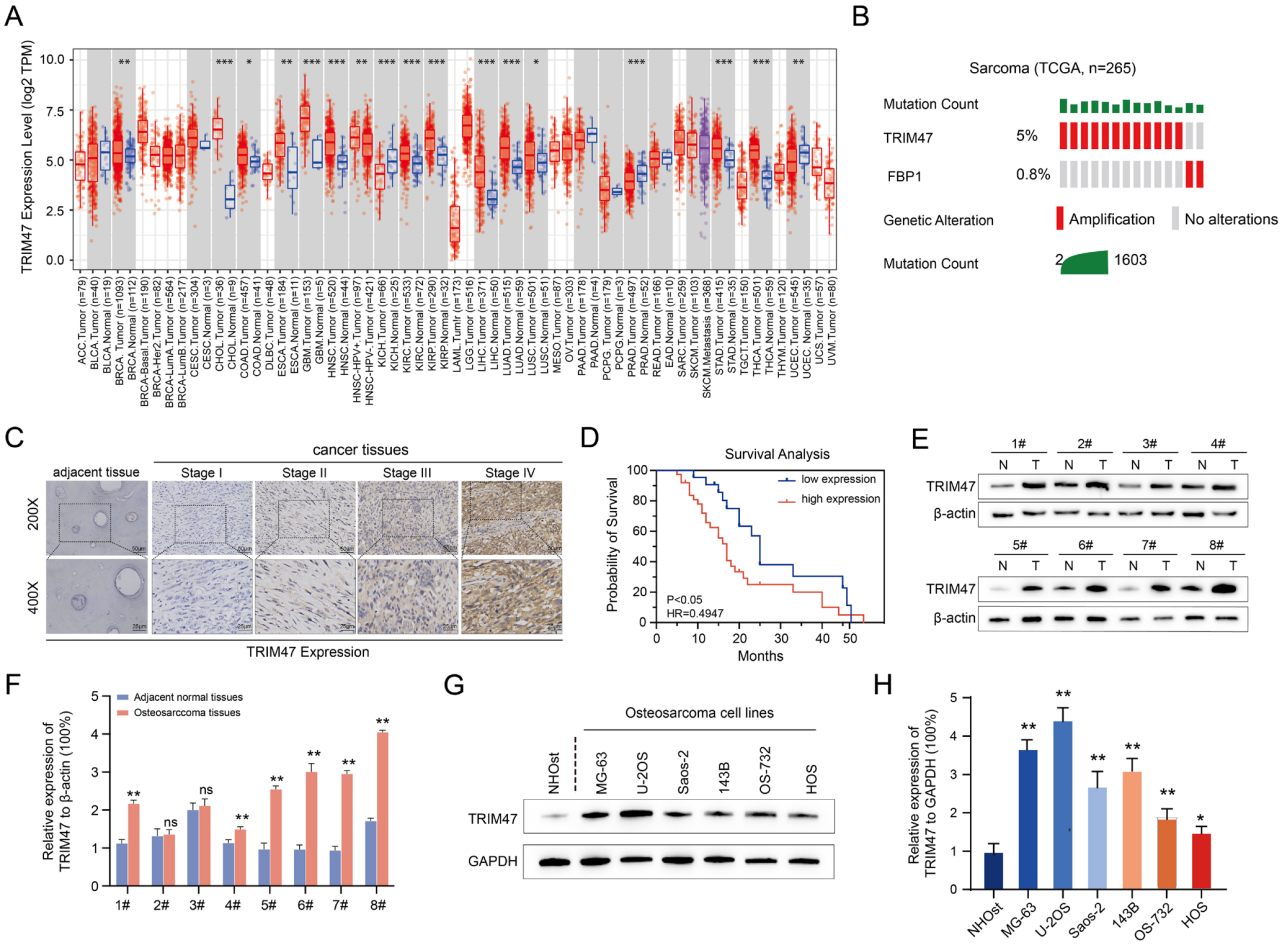
TRIM47 is frequently upregulated and positively associated with poor clinical outcomes of osteosarcoma patients. (A) Differential expression of TRIM47 in various tumours obtained from the TCGA database. (B) Bioinformatic analysis was performed to detect genetic alterations of TRIM47 in osteosarcoma tissues. (C) Immunohistochemical staining was performed to detect the TRIM47 protein expression in osteosarcoma specimens and corresponding adjacent non‐cancerous tissues. (D) Kaplan–Meier curve was plotted to explore the overall survival rate of enrolled osteosarcoma patients using the log‐rank test. (E) TRIM47 protein expression in 8 paired osteosarcoma tissues and adjacent normal tissues was analysed by western blotting assays. (F) Quantification of the protein expression levels of TRIM47 in osteosarcoma tissues analysed by Image J software. (G) Western blotting assays to examine the TRIM47 protein levels in osteosarcoma cell lines. (H) Bar graph of quantified relative TRIM47 expression normalised to GAPDH expression. Student's *t* test, *p < 0.05; **p < 0.01.

The expression levels of TRIM47 collected from sarcoma patients were analysed from the data collected from the online database (timer2.0, timer.comp‐genomics.org). Interestingly, TRIM47 expression was upregulated in a range of tumour types (Figure 1A), including sarcoma. Moreover, the mutation frequency of TRIM47 in osteosarcoma was up to 5% (Figure 1B). Therefore, we further confirmed the expression of TRIM47 in clinical specimens utilising IHC (immunohistochemistry) and western blotting analysis. As shown in Figure 1C, the immunostaining of TRIM47 in osteosarcoma tissues was much higher than that in adjacent non‐cancerous tissues. To further reveal the clinical significance of TRIM47 in osteosarcoma, the correlation of TRIM47 and clinicopathological features was evaluated. As illustrated in Table 1, we found that the expression of TRIM47 was positively related to the advanced clinical stage (*p* < 0.01) and distant lung metastasis (*p* = 0.021). Moreover, our survival analysis indicated that the overall survival of patients with high TRIM47 expression was much worse than that of those with low TRIM47 expression (Figure 1D, *p* < 0.05), indicating that TRIM47 may function as a prognostic marker for osteosarcoma patients. Consistently, the TRIM47 protein expression was found to be frequently overexpressed in eight fresh osteosarcoma tissues when compared with normal sarcoma tissues (Figure 1E, 6/8, 75%). Furthermore, the protein and mRNA expression of TRIM47 was also upregulated in osteosarcoma cell lines (Figure 1G,H). Taken together, TRIM47 is frequently upregulated and positively associated with poor clinical outcomes of osteosarcoma patients.

### 3.2 TRIM47 Knockdown Impedes Cell Proliferation and Invasion of Osteosarcoma Cells

**FIGURE 2:**
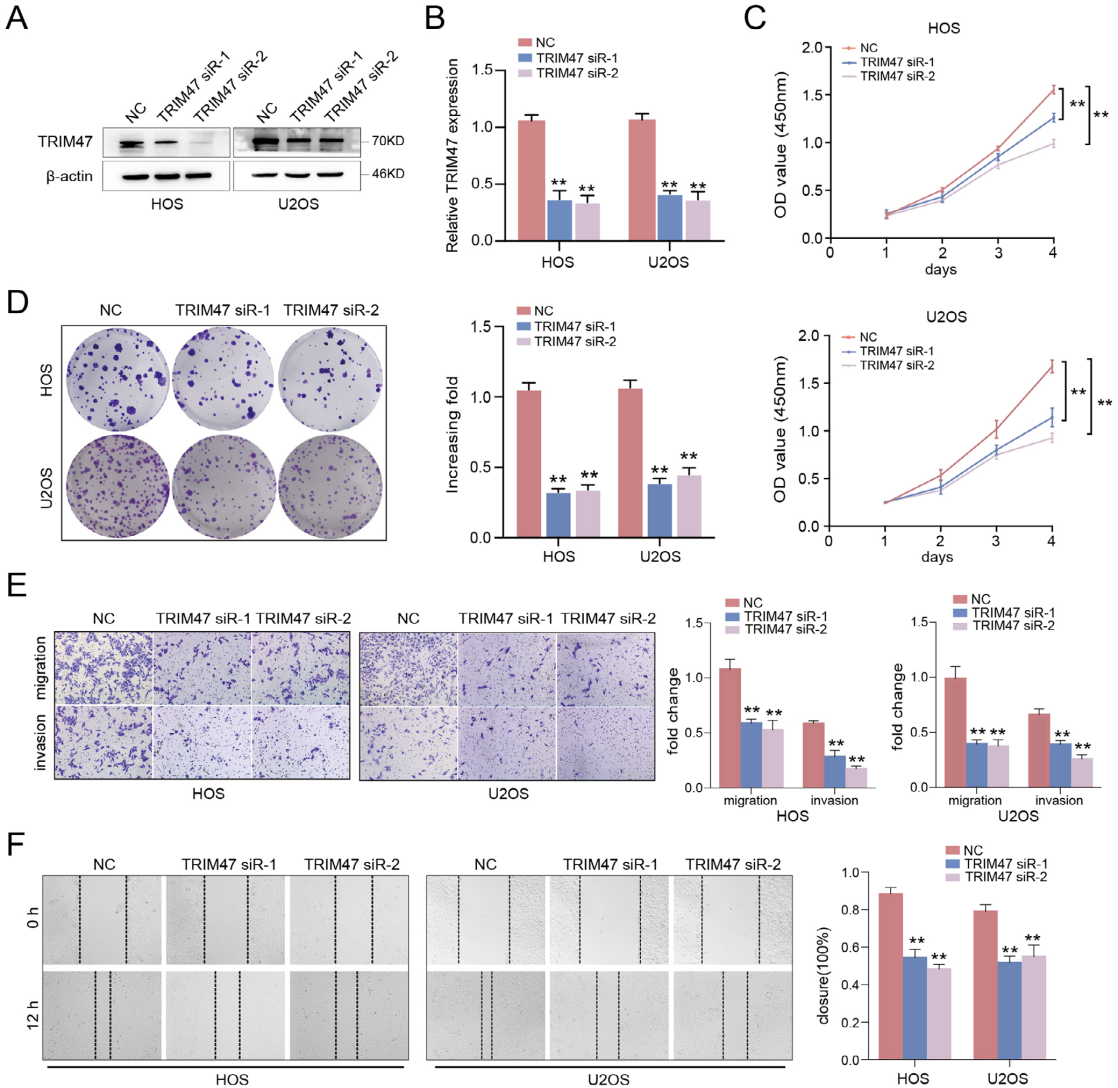
TRIM47 knockdown impedes cell proliferation and invasion of osteosarcoma cells. The transfection efficiency of TRIM47 siRNAs was confirmed by western blotting (A) and qRT‐PCR (B) experiments in osteosarcoma cells. (C) CCK‐8 assays were utilised to detect the cell viability upon TRIM47 depletion. (D) Representative photograph of colony forming assays for osteosarcoma cells after TRIM47 knockdown; the quantification of fold changes was shown in the bar graph. (E) Representative photographs and quantification of OS cells as indicated in transwell experiments (magnification 200×). (F) Wound healing assays were utilised to assess the migrative capacities of osteosarcoma cells. **p < 0.01 compared to the control cells.

Since TRIM47 might function as an aggressive biomarker of OS patients, we further investigated the cellular roles of TRIM47 in the malignant processes of osteosarcoma via knockdown experiments. We first verified the efficiency of transfection of our TRIM47 siRNAs by western blotting and qRT‐PCR assays (Figure 2A,B). We found that TRIM47 depletion suppressed the growth and colony formation abilities of HOS and U2OS cells as determined by CCK‐8 and colony formation assays (Figure 2C,D). To determine the effect of TRIM47 on the migratory and invasive ability of osteosarcoma cells, we utilised wound healing and transwell assays in osteosarcoma cells. Wound healing assays revealed that the closure rate in HOS and U2OS cells was decreased in the TRIM47 siRNA groups compared with the negative control (Figure 2E). Transwell migration assays revealed that TRIM47 depletion displayed a reduced migratory ability (Figure 2F). These data indicated that modulation of TRIM47 expression influenced the malignant behaviours of osteosarcoma cells in vitro.

### 3.3 TRIM47 Overexpression Facilitates the Aggressive Behaviours in Osteosarcoma Cells

**FIGURE 3:**
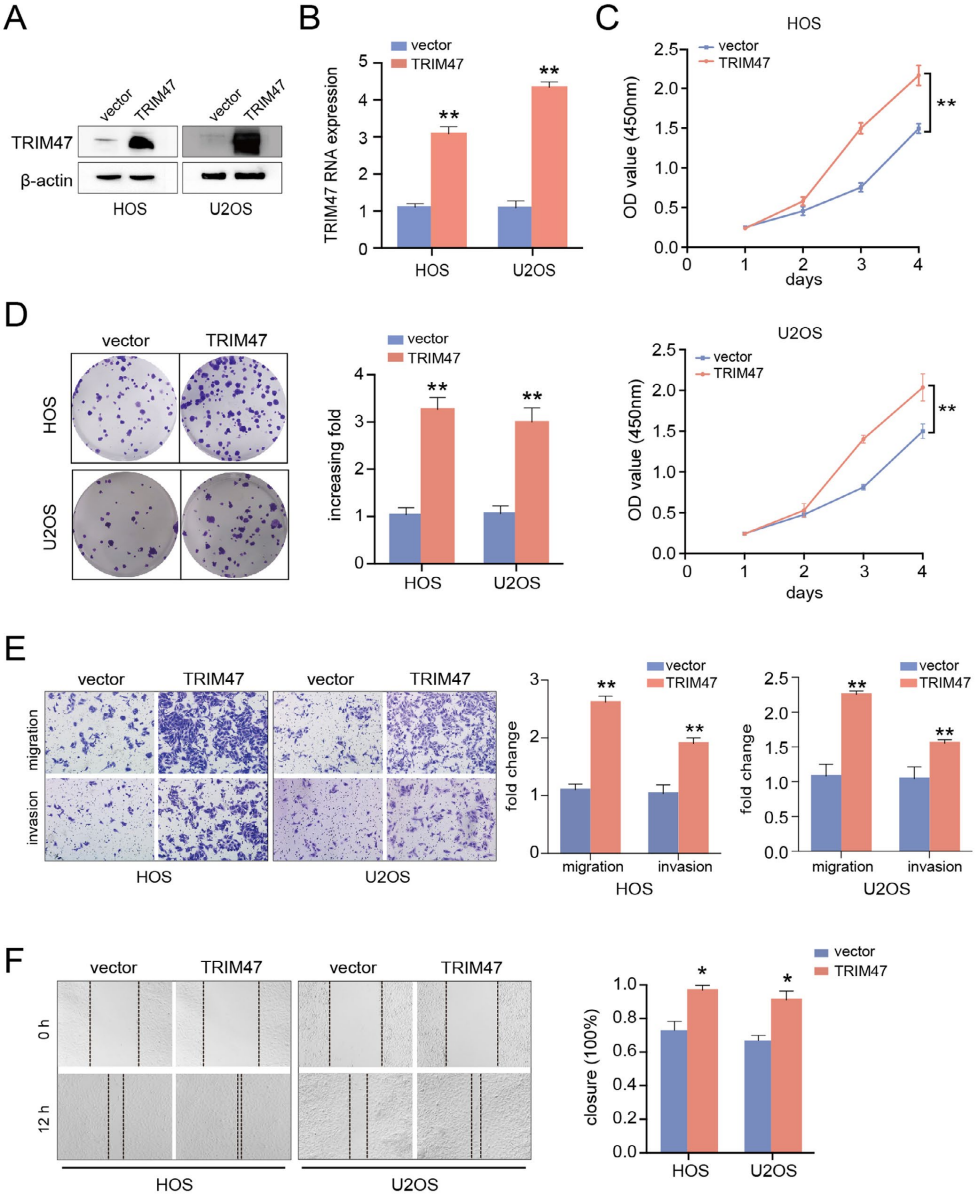
TRIM47 overexpression facilitates the aggressive behaviours in osteosarcoma cells. The transfection efficiency of TRIM47 overexpression was confirmed by western blotting (A) and qRT‐PCR experiments (B) in osteosarcoma cells. (C) CCK‐8 assays were utilised to detect the cell viability upon TRIM47 overexpression. (D) Colony formation (D), transwell invasion (E), and wound healing assays (F) were performed to evaluate the cell proliferation, invasion, and migration abilities of osteosarcoma cells upon TRIM47 overexpression. *p < 0.05, **p < 0.01 compared to the control cells.

We further investigated the cellular roles of TRIM47 via overexpression experiments. The transfection efficiency was confirmed by western blotting and qRT‐PCR assays (Figure 3A,B). Through cell proliferation (Figure 3C), colony formation (Figure 3D), migration and invasion (Figure 3E), and wound healing (Figure 3F) assays, we found that upregulation of TRIM47 promoted the proliferation, migration, and invasion of osteosarcoma cells. Collectively, these data demonstrate that TRIM47 is responsible for cancer cell proliferation, migration, and invasion in vitro and thus represents a potential therapeutic target for OS patients.

### 3.4 TRIM47 Triggers Wnt/β‐Catenin Pathway in Osteosarcoma Cells

**FIGURE 4:**
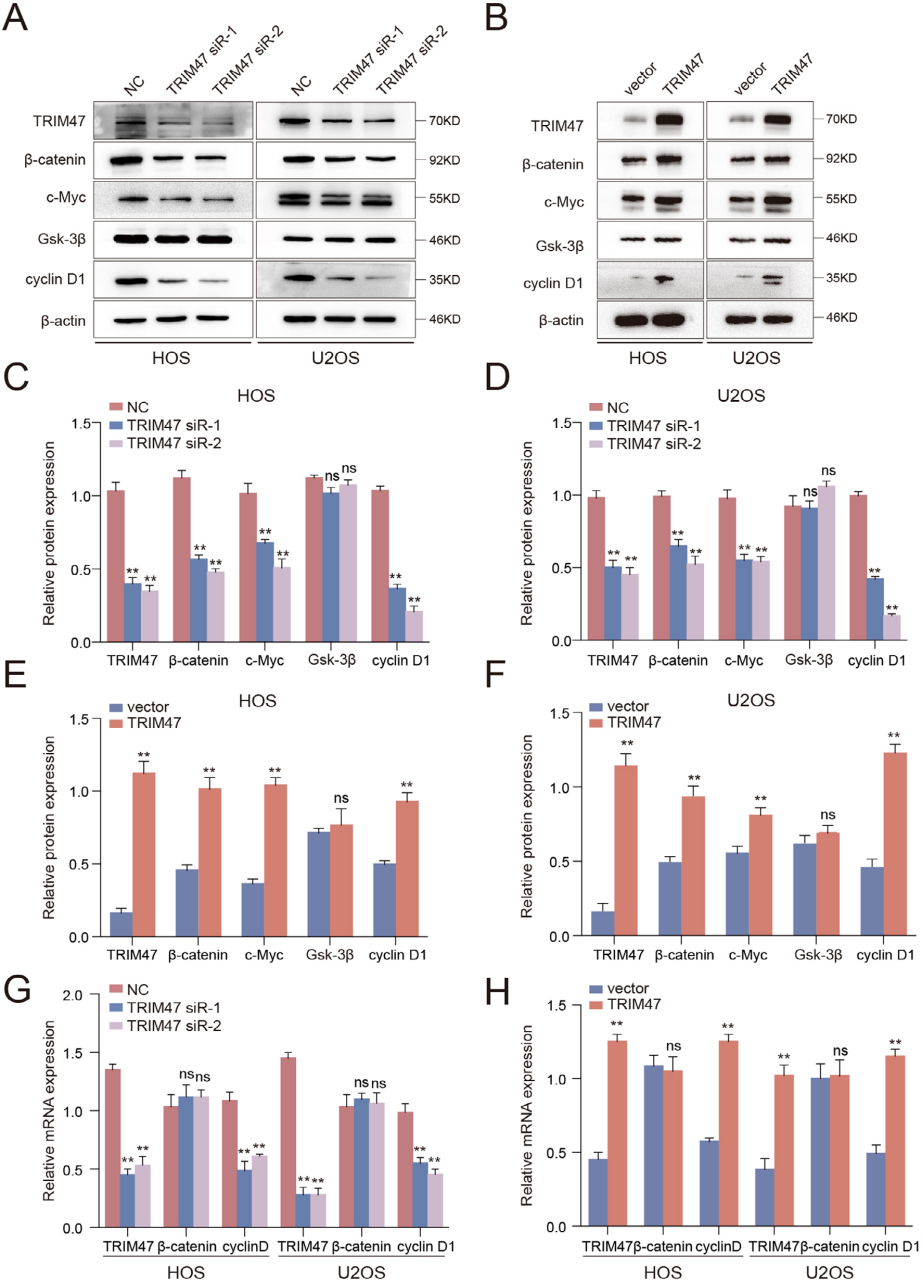
TRIM47 triggers the Wnt/β‐catenin pathway in osteosarcoma cells. Protein levels of TRIM47 and Wnt/β‐catenin cascade components were determined by western blotting assays in osteosarcoma cells upon TRIM47 knockdown (A) or upregulation (B). (C–F) Quantitative analysis of protein levels of TRIM47 and Wnt pathway components in HOS and U2OS cells with TRIM47 knockdown or overexpression. (G and H) The mRNA levels of Wnt/β‐catenin pathway components were detected by qRT‐PCR assays upon indicated treatments in osteosarcoma cells. All these experiments were repeated at least twice. ns, no significance; **p < 0.01.

Wnt/β‐catenin pathway is one of the predominant oncogenic signaling pathways accounting for tumorigenesis and progression, including osteosarcoma [19, 25, 26]. A recent study has demonstrated that knockdown of TRIM47 suppressed cell proliferation and metastasis of glioma cells via negatively regulating Wnt signalling [27]; therefore, we explored whether the Wnt/β‐catenin pathway was also accountable for TRIM47 function in osteosarcoma. Strikingly, in this study, we found that β‐catenin was significantly decreased upon TRIM47 interference, resulting in the reduction of c‐Myc and cyclin D1, several well‐documented target genes of the Wnt/β‐catenin pathway (Figure 4A,C,E). On the contrary, overexpression of TRIM47 revealed the opposite effects in osteosarcoma cells (Figure 4B,D,F). However, qRT‐PCR detection found no significant changes in β‐catenin at the mRNA level after modulation of TRIM47 expression (Figure 4G,H), indicating that TRIM47 regulates the Wnt/β‐catenin pathway through post‐transcriptional or translational modification. The above results suggest that TRIM47 positively triggers the Wnt/β‐catenin signalling pathway in osteosarcoma cells.

### 3.5 TRIM47 Binds to and Ubiquitinates FBP1 Protein

**FIGURE 5:**
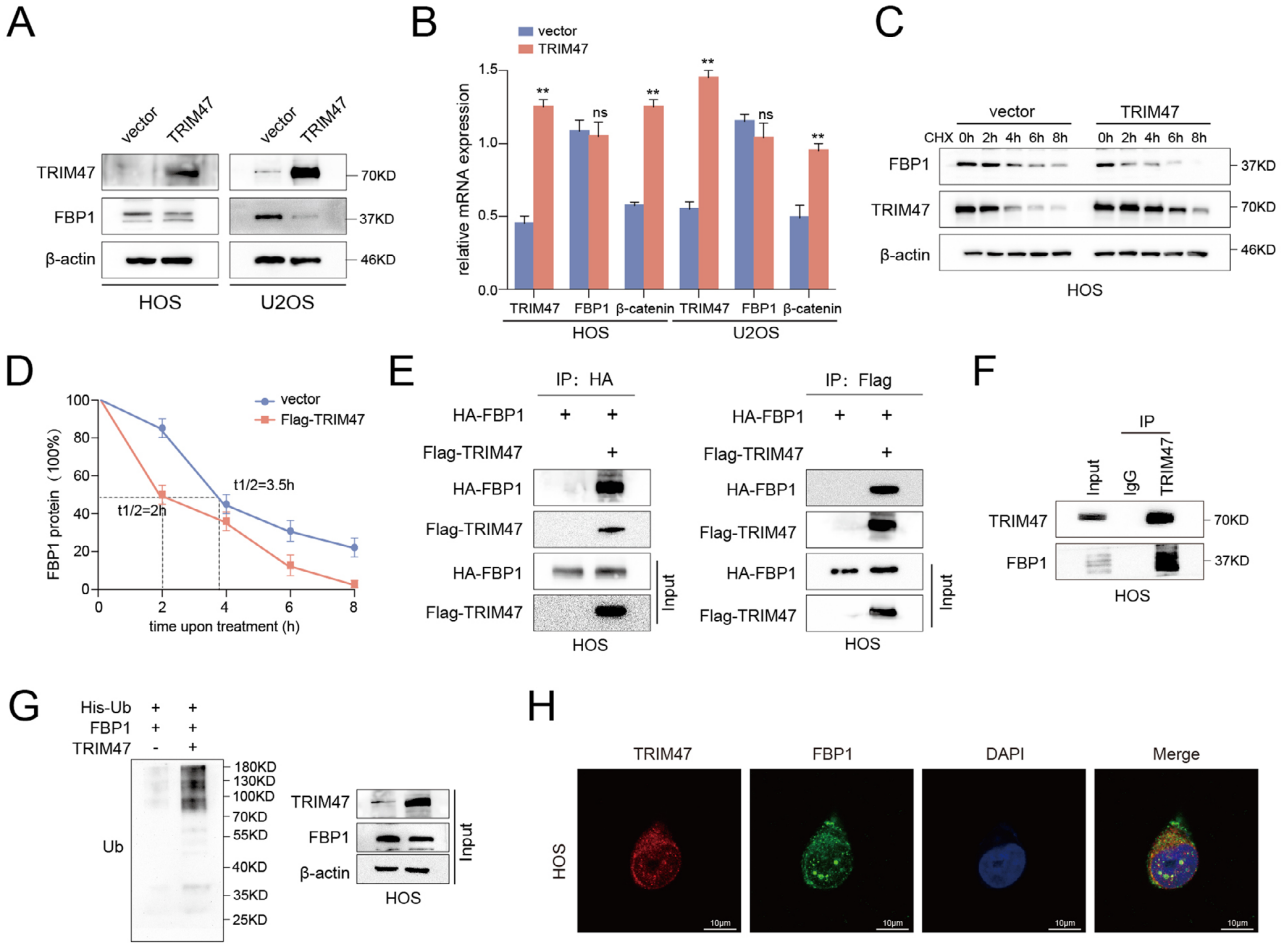
TRIM47 binds to and ubiquitinates the FBP1 protein. The expression of FBP1 protein after overexpressing TRIM47 was confirmed by western blotting (A) and qRT‐PCR experiments (B) in osteosarcoma cells. (C) Representative western blotting images of FBP1 protein in HOS cells added with 100 μg/mL cycloheximide (CHX) for 0, 2, 4, 6, and 8 h. (D) The line graph was plotted to reveal FBP1 expression over time, normalised to β‐actin controls. (E) Co‐immunoprecipitation (Co‐IP) experiments were performed to observe the interaction between TRIM47 and FBP1 protein in osteosarcoma HOS cells. (F) The HOS cell lysates were immunoprecipitated with anti‐TRIM47 or control immunoglobulin G (IgG) to detect the interaction between endogenous TRIM47 and FBP1. (G) The in vivo ubiquitination assays were performed to detect the FBP1 protein expression upon TRIM47 overexpression. (H) Immunofluorescence staining was performed to determine the subcellular localisation of TRIM47 (red) and FBP1 (green) in HOS cells. Cell nuclei were counterstained with DAPI blue for nuclear visualisation. **p < 0.01 compared to the control cells.

To identify potential downstream substrates of the E3 ubiquitin ligase TRIM47, we screened the literature and found that the ubiquitination of FBP1 by TRIM47 was involved in the carcinogenesis of pancreatic cancer [28]. FBP1 (fructose 1, 6‐bisphosphatase 1), a rate‐limiting enzyme in gluconeogenesis, was reported to be downregulated in various tumours, including OS [29]. However, the detailed molecular mechanism underlying the involvement of TRIM47 and FBP1 in OS cells still needs to be further explored. As we suspected, the protein expression of FBP1 was noticeably reduced upon TRIM47 overexpression (Figure 5A). Moreover, qRT‐PCR data showed that the FBP1 mRNA was not significantly changed upon TRIM47 overexpression (Figure 5B), indicating the regulation of FBP1 protein by TRIM47 was at post‐transcriptional levels. To confirm our hypothesis, we further performed the cycloheximide (CHX) chase assays to test the modulation of the half‐life of FBP1 protein by TRIM47 upregulation. As we suspected, the half‐life of FBP1 protein was markedly shortened after TRIM47 overexpression (3.5 h vs. 2 h, *p* < 0.05, Figure 5C,D). As a matter of fact, the co‐IP assays indicated that TRIM47 could bind to FBP1 both exogenously and endogenously (Figure 5E,F). Furthermore, we also found that overexpression of TRIM47 led to an induction in the ubiquitination level of FBP1 protein (Figure 5G). Additionally, the co‐immunofluorescent analysis showed that TRIM47 and FBP1 were mainly co‐localized in the cytoplasm. Summarily, these findings indicate that TRIM47 interacts and destabilises FBP1 protein via ubiquitination.

### 3.6 TRIM47 Enhances the Proliferation, Migration, and Invasion of Osteosarcoma Cells by Regulating FBP1 and Activating the Wnt/β‐Catenin Pathway

**FIGURE 6:**
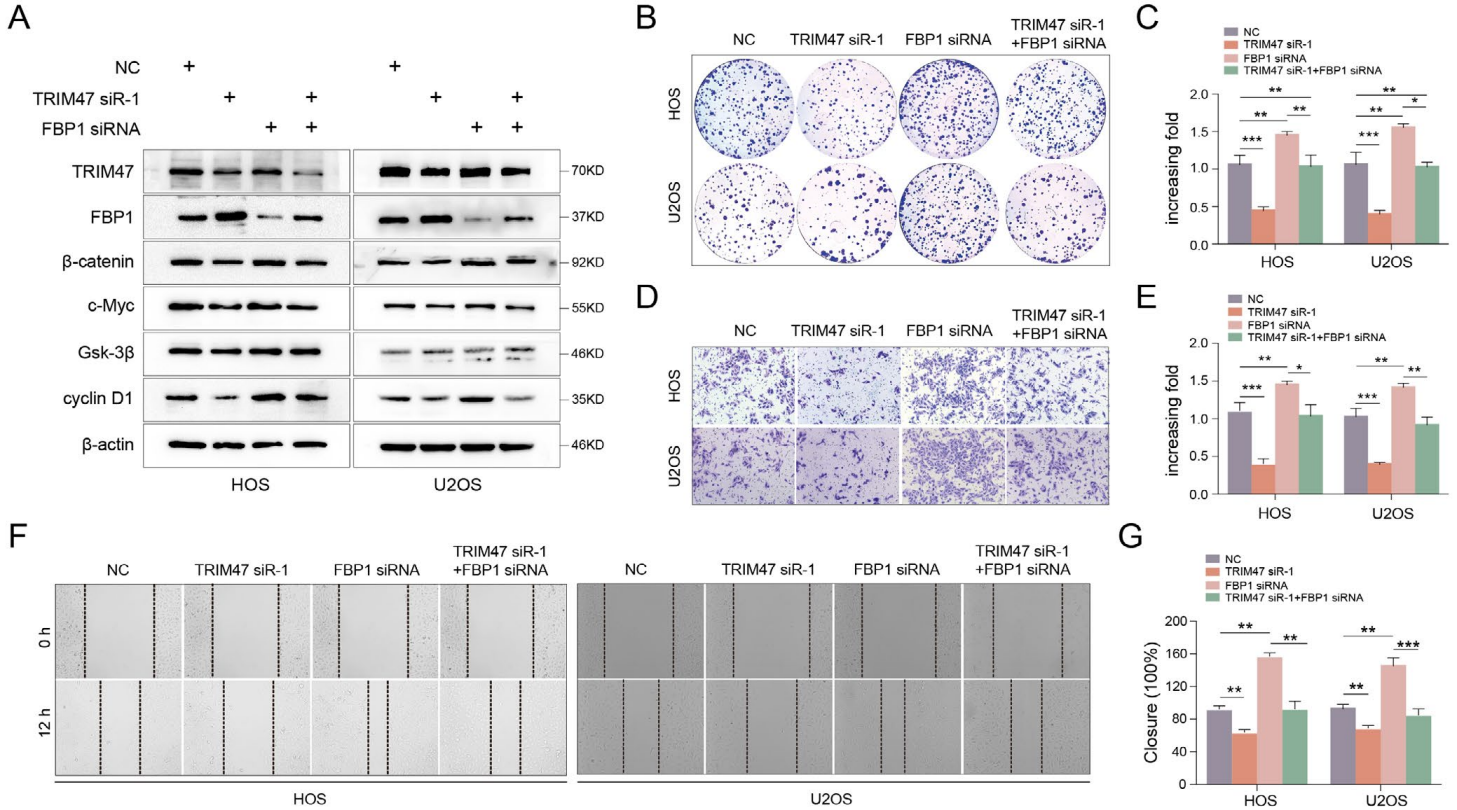
TRIM47 enhances the proliferation, migration, and invasion of osteosarcoma cells by regulating FBP1 and activating the Wnt/β‐catenin pathway. (A) OS cells were transfected with TRIM47 siRNA and FBP1 siRNA together or separately. Western blotting analysis was utilised to detect the protein levels of TRIM47, FBP1, and Wnt/β‐catenin core components. The proliferation, invasive, and migrative abilities of U2OS and HOS cells were determined by colony formation (B, C), transwell invasion (D, E), and wound healing assays (F, G). *p < 0.05, **p < 0.01 compared to the controls.

A previously published literature has demonstrated that forced overexpression of FBP1 inhibits proliferation and metastasis in cholangiocarcinoma cells via the Wnt/β‐catenin pathway [30]; therefore, we suspected that TRIM47 might regulate the malignant phenotypes of osteosarcoma cells through modulation of FBP1‐Wnt/β‐catenin signalling. Thus, we performed the knockdown of TRIM47 and FBP1 individually and in combination in HOS and U2OS cells. As shown in Figure 6A, FBP1 depletion partially abolished the downregulation of β‐catenin, c‐Myc, and cyclin D1 mediated by TRIM47 knockdown. Likewise, FBP1 deficiency significantly abrogated the suppression of colony formation abilities caused by TRIM47 depletion (Figure 6B,C). Similarly, the wound healing and transwell invasion assays revealed that FBP1 downregulation recovered the suppressive role of TRIM47 knockdown on migrative and invasive abilities in U2OS and HOS cells (Figure 6D–G, *p* < 0.05). More convincingly, we found that when TRIM47 and FBP1 overexpression plasmids were co‐transfected, the overexpression of FBP1 rescued the effects of TRIM47 in OS cells (Figure S1), which further supported that FBP1 functions as a crucial mediator of TRIM47 to promote osteosarcoma cell proliferation and invasion by regulating the Wnt/β‐catenin pathway. Taken together, these data reveal that the FBP1‐Wnt/β‐catenin axis is critical for the effects of TRIM47 in facilitating the progression of human OS cells.

### 3.7 TRIM47 Depletion Impeded Osteosarcoma Tumourigenesis In Vivo

**FIGURE 7:**
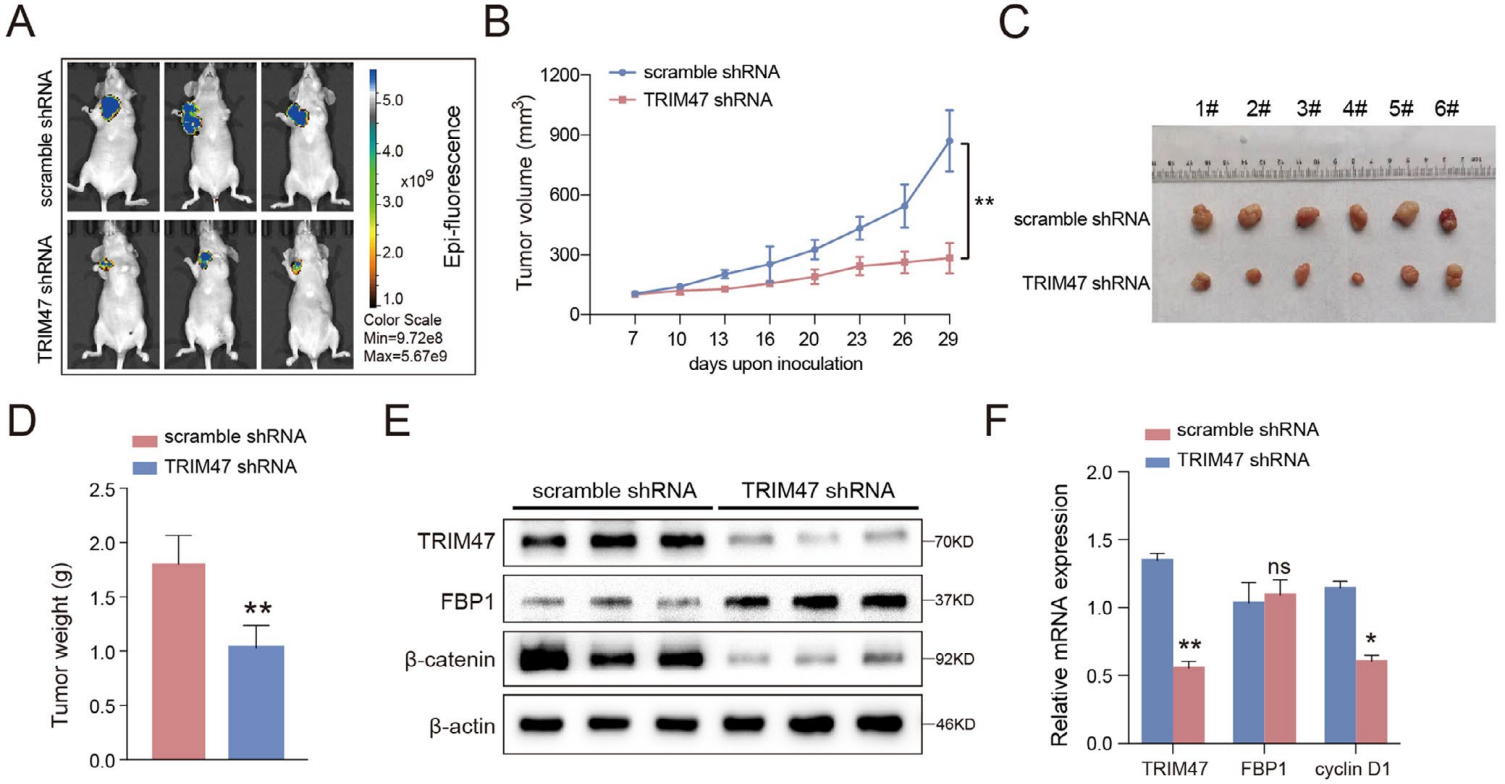
TRIM47 depletion impeded osteosarcoma tumorigenesis in vivo. (A) Representative pictures of xenograft tumours. (B) Tumour volume growth curve of the mice; the tumour volume was calculated according to the formula (L × W^2^)/2. (C) Images of xenograft tumours harvested on the 29th day. (D) The tumour weights of the tumours obtained from the two groups are shown in a bar graph. (E) The protein levels of TRIM47, FBP1, and Wnt pathway core components were analysed in xenograft tumours. (F) The mRNA expression levels of TRIM47, FBP1, and cyclin D1 in the six harvested tumours were detected by qRT‐PCR assays. ns, no significance, *p < 0.05, **p < 0.01.

**FIGURE 8:**
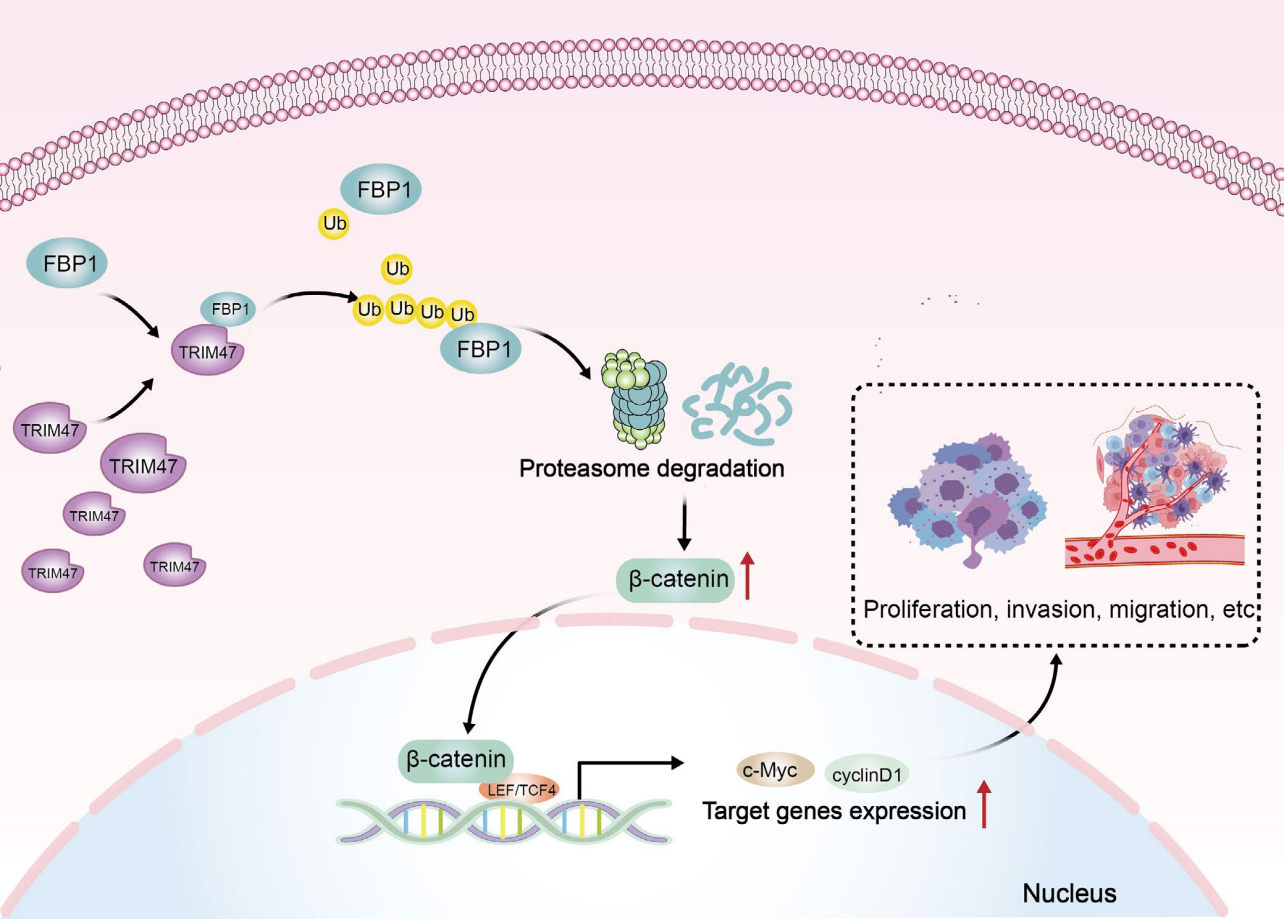
A model of TRIM47 regulation of FBP1‐Wnt/β‐catenin pathway in osteosarcoma.

To elucidate the impact of TRIM47 on osteosarcoma carcinogenesis more credibly, we constructed the subcutaneous tumour formation model to evaluate the tumour cell growth in vivo (Figure 7A). As shown in Figure 7B, TRIM47 knockdown dramatically repressed tumour growth. Moreover, the xenograft tumours in the TRIM47 downregulation groups were much lighter than those in the control groups (Figure 7C,D). Consistent with our previous cellular experiments, TRIM47 depletion significantly upregulated FBP1 protein expression, thereby suppressing the Wnt/β‐catenin signalling pathway (Figure 7E). Furthermore, the mRNA expression levels of TRIM47 and Wnt pathway target cyclin D1 were also markedly reduced, along with no significant changes in FBP1 mRNA expression upon TRIM47 depletion (Figure 7F). Collectively, TRIM47 knockdown impeded the tumour growth of osteosarcoma cells via regulating the FBP1‐β‐catenin axis in vivo. We also constructed a simple diagram to illustrate the process by which TRIM47 regulates FBP1 to promote the malignant progression of OS (Figure 8).

## 4 Discussion

An increasing body of evidence has highlighted that TRIM family proteins play critical roles in carcinogenesis and cancer progression [9, 10, 31, 32]. In this study, we unveiled the clinical significance, biological functions, as well as underlying mechanisms of TRIM47 in osteosarcoma carcinogenesis and progression. TRIM47 was drastically overexpressed, positively correlated with clinical pathological grade, and poor prognosis in osteosarcoma patients. Moreover, TRIM47 was essential in sustaining cell proliferation, migration, and invasion in osteosarcoma cells. Regarding the underlying mechanisms, we firstly evidenced that TRIM47 interacted and destabilised FBP1 protein, thus facilitating the activation of the Wnt/β‐catenin pathway, consequently leading to tumorigenesis and development in OS cells.

Posttranslational modification (PTM) is a well‐documented regulatory mechanism of proteins, accounting for the occurrence and development of several tumours [33]. Ubiquitination is an important pattern of PTM, playing a significant role in OS recurrence and metastasis [17, 34]. Recently, TRIM47, a member of the E3 ubiquitin ligase, has been upregulated in a series of human cancers, such as gastric cancer, breast cancer, and hepatocellular carcinoma [11, 12, 14]. TRIM47 was frequently overexpressed and positively correlated with poor prognosis in non‐small cell lung cancer [13]. Additionally, TRIM47 confers PARP inhibitor sensitivity via BRCA1 ubiquitination in triple‐negative breast cancer cells [32]. Here, we found that TRIM47 was frequently upregulated in osteosarcoma tissues. In addition, the TRIM47 upregulation was positively associated with aggressive clinical stage and worse overall survival, indicating its role as a prognostic marker in osteosarcoma. Furthermore, we demonstrated that TRIM47 depletion significantly impeded the cell growth and invasion capacities, which were promoted by TRIM47 overexpression. These findings further indicate that TRIM47 harbours oncogenic potential, suggesting that targeting TRIM47 may provide candidate therapeutic targets for osteosarcoma patients in the future. However, the molecular mechanisms by which TRIM47 exerts oncogenic roles in osteosarcoma still need to be further investigated.

It has been well established that the Wnt/β‐catenin pathway is involved in osteosarcoma oncogenesis [25, 26]. Wang et al. [12] have demonstrated that TRIM47 knockdown exerts cancer‐suppressing functions via suppressing the Wnt/β‐catenin pathway. However, the detailed interplays linking TRIM47 and Wnt/β‐catenin in osteosarcoma remain uninvestigated. In the present research, we firstly identified that TRIM47 served as a novel positive regulator of the Wnt/β‐catenin pathway in osteosarcoma. TRIM47 overexpression increased the protein and mRNA expression of the Wnt pathway target gene (including c‐Myc and cyclin D1), indicating that β‐catenin was activated in the nucleus upon TRIM47 upregulation. As a member of the E3 ubiquitin ligase, TRIM47 predominantly exerts its biological functions by destabilising and ubiquitinating downstream targets [32]. For example, TRIM47 facilitates breast cancer proliferation and endocrine therapy resistance by stabilising PKC‐ε/PKD3 [35]. Additionally, TRIM47 facilitates K48‐linked ubiquitination, leading to a decrease in CDO1 protein abundance in HCC. FBP1 (fructose‐1,6‐biphosphatase), a rate‐limiting enzyme in gluconeogenesis, served as a negative modulator of the Wnt/β‐catenin pathway [32], while the upstream regulators mediating the FBP1‐Wnt/β‐catenin axis were unclear. Herein, we identified FBP1 as the downstream substrate of TRIM47 in OS cells, while the biological functions of FBP1 in OS remain unclear. In this study, we found that the knockdown of FBP1 impeded cell proliferation and invasion via suppressing Wnt//β‐catenin pathway in OS cells. Mechanistically, TRIM47 interacted with FBP1 and promoted its degradation in a ubiquitination‐dependent manner. However, the possible binding domains between TRIM47 and FBP1 proteins need further exploration in our future study.

More intriguingly, in vitro functional assays indicated that depletion of FBP1 could reverse the decreased malignant behaviours in TRIM47‐downregulated cells. However, the inhibitory effects of TRIM47 knockdown could not be wholly abolished by FBP1 knockdown, indicating that FBP1 is not the unique gate linking TRIM47 to the Wnt/β‐catenin pathway. Recent studies have reported that TRIM47 accelerates malignant progression through promoting the ubiquitination and degradation of critical tumour suppressors, such as p53, BRCA1, and SMAD4 [15, 32, 36]. Additionally, PI3K/Akt and NF‐κB pathways were also responsible for TRIM47 functions in breast cancer [12, 16]. Thus, we suspect that the above‐mentioned regulatory networks may also participate in TRIM47‐mediated carcinogenesis.

## 5 Conclusions

In summary, our study unveils TRIM47 as a bona fide activator of the Wnt/β‐catenin pathway to facilitate osteosarcoma tumourigenesis and progression via ubiquitinating FBP1 protein.

## Author Contributions

**Heng Wang:** formal analysis (equal), funding acquisition (supporting), resources (equal), writing – original draft (equal). **Xiao Chen:** data curation (supporting), formal analysis (supporting), software (supporting), validation (supporting). **Fengting Nie:** data curation (equal), formal analysis (equal), writing – original draft (equal). **Min Zhong:** methodology (equal), project administration (equal), resources (equal), software (equal), validation (equal). **Zhi Fang:** visualization (equal), writing – original draft (equal). **Zezhi Qiu:** data curation (supporting). **Ling Zhou:** investigation (supporting), methodology (supporting), resources (supporting), supervision (supporting), visualization (supporting). **Yi Le:** conceptualization (supporting), data curation (supporting), software (supporting), validation (supporting). **Xianpin Wei:** data curation (supporting), formal analysis (equal), resources (supporting), software (supporting). **Yanyu Liao:** data curation (supporting), investigation (supporting), methodology (supporting). **Ziling Fang:** conceptualization (equal), funding acquisition (equal), methodology (equal), project administration (equal), writing – review and editing (equal).

## Conflicts of Interest

The authors declare no conflicts of interest.

## Supporting information


Figure S1.

Table S1.

## Data Availability

The datasets performed or analysed in this work are available from the corresponding author Ziling Fang, upon reasonable request.
