# Supplementary material for: TRIM47 Facilitates Osteosarcoma Progression via Destabilising FBP1 and Thus Activation of Wnt/β‐Catenin Pathway
Source: J Cell Mol Med. 2025 Aug 13;29(15):e70753. doi: 10.1111/jcmm.70753 (PMC12344859; doi:10.1111/jcmm.70753)

**Figure S1 FBP1 rescues the oncogenic effects of TRIM47 overexpression.**

**(A)** OS cells were transfected with TRIM47 overexpression and FBP1 overexpression together or separately. Western blotting analysis was utilized to detect the protein levels of TRIM47, FBP1 and Wnt/β-catenin core components. The proliferation and invasive abilities of U2OS and HOS cells were determined by colony formation **(B, C)** and transwell invasion **(D, E)** assays. *p< 0.05, **p< 0.01 compared to the indicated controls.


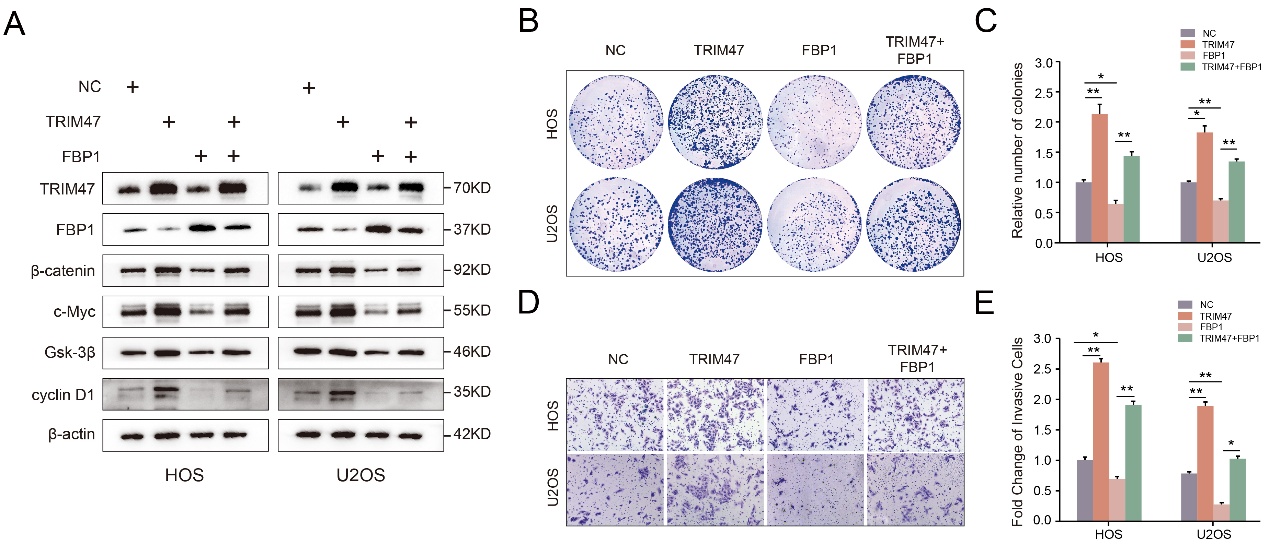

Supplement: Supplementary file 1 — Figure S1. [file JCMM-29-e70753-s001.docx]
