# Supplementary material for: TRIM47 Facilitates Osteosarcoma Progression via Destabilising FBP1 and Thus Activation of Wnt/β‐Catenin Pathway
Source: J Cell Mol Med. 2025 Aug 13;29(15):e70753. doi: 10.1111/jcmm.70753 (PMC12344859; doi:10.1111/jcmm.70753)
Supplement: Supplementary file 2 — Table S1. [file JCMM-29-e70753-s002.docx]

**Supplementary Table 1. The clinicopathological characteristics of osteosarcoma patients.**

| **Patients** | **Age (year)** | **gender** | **Tumor size (cm)** | **Size of tumor invasion** | **Differentiation**  **(well, moderate and poorly)** | **AJCC stage** | **Lymph node metastasis**  **(N_0_ or N_X_)** |
| --- | --- | --- | --- | --- | --- | --- | --- |
| #1 | 12 | Male | 6*2.5*0.3 | T_1_ | moderate | IIA | N_0_ |
| #2 | 50 | Male | 9.5*2.5*4 | T_2_ | moderate | IIB | N_0_ |
| #3 | 35 | Male | 15*11*6.5 | T_2_ | well | IIB | N_X_ |
| #4 | 50 | Female | 10.2*8.3*5 | T_2_ | well | IIB | N_0_ |
| #5 | 28 | Male | 6*4.5*1.8 | T_1_ | moderate | IIA | N_X_ |
| #6 | 56 | Male | 7.5*4*3 | T_2_ | moderate | IV | N_0_ |
| #7 | 23 | Male | 4.5*4.5*2.5 | T_1_ | moderate | IIA | N_X_ |
| #8 | 14 | Male | 2.5*1.5*0.4 | T_1_ | moderate | IIA | N_0_ |
